# Association between Maternal Perinatal Stress and Depression on Infant DNA Methylation in the First Year of Life

**DOI:** 10.21203/rs.3.rs-3962429/v1

**Published:** 2024-03-21

**Authors:** Sarina Abrishamcar, Beryl Zhuang, Mara Thomas, Nicole Gladish, Julia MacIsaac, Meaghan Jones, Elinor Simons, Theo Moraes, Piush Mandhane, Jeffrey Brook, Padmaja Subbarao, Stuart Turvey, Edith Chen, Gregory Miller, Michael Kobor, Anke Huels

**Affiliations:** Emory University; University of Manitoba; University of Manitoba; Northwestern University; Northwestern University; Rollins School of Public Health, Emory University

## Abstract

Maternal stress and depression during pregnancy and the first year of the infant’s life affect a large percentage of mothers. Maternal stress and depression have been associated with adverse fetal and childhood outcomes as well as differential child DNA methylation (DNAm). However, the biological mechanisms connecting maternal stress and depression to poor health outcomes in children are still largely unknown. Here we aim to determine whether prenatal stress and depression are associated with changes in cord blood mononuclear cell DNAm (CBMC-DNAm) in newborns (n = 119) and whether postnatal stress and depression are associated with changes in peripheral blood mononuclear cell DNAm (PBMC-DNAm) in children of 12 months of age (n = 113) from the Canadian Healthy Infant Longitudinal Development (CHILD) cohort. Stress was measured using the 10-item Perceived Stress Scale (PSS) and depression was measured using the Center for Epidemiologic Studies Depression Questionnaire (CESD). Both stress and depression were measured at 18 weeks and 36 weeks of pregnancy and six months and 12 months postpartum. We conducted epigenome-wide association studies (EWAS) using robust linear regression followed by a sensitivity analysis in which we bias-adjusted for inflation and unmeasured confounding using the bacon and cate methods. To investigate the cumulative effect of maternal stress and depression, we created composite prenatal and postnatal adversity scores. We identified a significant association between prenatal stress and differential CBMC-DNAm at 8 CpG sites and between prenatal depression and differential CBMC-DNAm at 2 CpG sites. Additionally, we identified a significant association between postnatal stress and differential PBMC-DNAm at 8 CpG sites and between postnatal depression and differential PBMC-DNAm at 11 CpG sites. Using our composite scores, we further identified 2 CpG sites significantly associated with prenatal adversity and 7 CpG sites significantly associated with postnatal adversity. Several of the associated genes, including *PLAGL1, HYMAI, BRD2*, and *ERC2* have been implicated in adverse fetal outcomes and neuropsychiatric disorders. This suggested that differential DNAm may play a role in the relationship between maternal mental health and child health.

## Introduction

Psychosocial factors such as maternal stress and depression during pregnancy and the first year of the infants’ life affect a large percentage of mothers (pre- and postnatal depression: ~12%; pre- and postnatal stress: ~25%^1,2^). Gestation and the first years of life are critical and particularly sensitive periods for child development^3^. Psychosocial stress during this time can affect the developmental trajectory of the child and increase susceptibility to adverse fetal and childhood outcomes^4–7^.Maternal stress and depression during pregnancy have been associated with adverse fetal outcomes such as low birth weight and preterm birth^8,9^ as well as adverse childhood outcomes^10–15^. Prenatal stress has been previously associated with neurodevelopmental disorders in children, including an increased risk of attention deficit hyperactivity disorder (ADHD), autism spectrum disorder (ASD), cognitive delay, and schizophrenia^10–12^. Prenatal depression has been associated with variations in white matter integrity, with implications for emotional and behavioral function, cognitive development, language development, and motor development in children^13–15^. Mother-infant interactions during the first year of life play a major role in the behavioral and cognitive development of the child^16–18^. Maternal postpartum stress or depression during this time has been associated with behavioral dysfunction (anger, withdrawal) and delayed cognitive development in infants^19,20^. Additionally, infants may express abnormal attachment patterns in response to maternal disengagement^21^.

While maternal stress and depression during the perinatal period have been linked to the etiology of a range of health outcomes in children, the biological mechanisms are still unknown. In the prenatal period, it has been hypothesized that maternal stress and depression affect the fetus through the mother’s hypothalamic-pituitary-adrenal (HPA) axis, which produces cortisol in response to stressors^22^. Cortisol, a glucocorticoid (GC) hormone colloquially known as the “stress hormone”, can cross the placenta and shape fetal development^23^. While physiological concentrations of GCs are critical for fetal brain development, excess exposure to GCs can be neurotoxic and negatively affect fetal development^24^. Other biological pathways have also been proposed, including catecholamines, oxidative stress, pro-inflammatory cytokines, serotonin, and the maternal gut-brain axis^6^. Postnatally, it has been hypothesized that cortisol may be passed to the child via breastmilk which can in turn affect child development^25^. However, postpartum maternal stress and depression seem to manifest in the child as behavioral and cognitive dysfunction even after accounting for breastfeeding status^26,27^. At the biological level, high stress and depression levels may induce epigenetic modifications which in turn can increase the production of maternal cortisol and other potential biological mediators^6,28,29^. Epigenetic modifications, such as differential DNA methylation (DNAm) are malleable and sensitive to psychosocial factors^30^. Epigenetic regulation plays an important role in cell differentiation and development, as well as in mediating adaptive responses to psychosocial influences. Additionally, DNAm is potentially reversible, suggesting that the methylome could be a therapeutic target for disease treatment and prevention^31^.

Despite widespread interest in the role of epigenetics as a potential nexus between the mother and the child, the literature on maternal psychosocial stress and child DNA methylation is incomplete and contradictory. Several epigenome-wide association studies (EWAS) have identified associations between prenatal depression and differential cord blood DNAm^32–35^,while only a few EWAS have investigated the associations between prenatal stress and DNA methylation, and the associations they report are inconsistent. For example, one study found null associations between prenatal perceived stress and cord blood DNAm^36^. Another study did find an association between prenatal perceived stress and neonatal saliva DNAm using a prenatal distress questionnaire and cortisol as measures of prenatal stress^37^. Meta-analyses of large-scale EWAS have also been inconsistent in their findings regarding the nature and strength of the associations between prenatal stress, depression, and variations in DNAm^38–41^. In contrast, several candidate gene studies have investigated differential DNAm in genes such as *11β-HSD2, FKBP5, and NR3C1* as potential mediating factors^40,42,43^. For example, differential DNAm in *11β-HSD2* and *NR3C1* and their respective interaction with prenatal depression have been associated with adverse neurodevelopmental outcomes in children^42^. During the postnatal period, associations between postpartum stress and depression and child DNA methylation have not been extensively studied despite the established relationship between postpartum maternal mental health and child development. While evidence of an association between postpartum depressive symptoms and numerous differentially methylated gene regions have been identified in two studies using next-generation sequencing^44^ and the Illumina EPIC array^45^ respectively, more EWASs, candidate gene studies, and large-scale meta-analyses have yet to be conducted.

Here, we conducted a prospective EWAS of maternal psychosocial status and child DNAm in a study of 131 children from the Canadian Healthy Infant Longitudinal Development (CHILD) cohort, using well-established measures of stress and depression^46^. Specifically, we investigated whether prenatal stress and depression are associated with variations in cord-blood mononuclear cell DNAm (CBMC-DNAm) in newborns and whether postnatal stress and depression are associated with changes in peripheral blood mononuclear cell DNAm (PBMC-DNAm) among one-year-olds. Stress and depression were analyzed separately, as well as in combination using composite adversity scores to explore the cumulative effect of stress and depression on DNAm. Additionally, to validate the robustness of our findings across exposure time points, we 1) investigated the associations between prenatal stress and depression and infant DNA methylation (PBMC-DNAm) at 12 months of age and 2) adjusted for prenatal stress in the postnatal stress model and for prenatal depression in the postnatal depression model to control for possible confounding by prenatal exposure.

## Materials and Methods

### Study population

The CHILD cohort is a Canadian population-based birth cohort^46,47^. Mothers (N = 3624) were enrolled during their second trimester of pregnancy between 2008 and 2012 and followed through pregnancy at four sites in Canada (Vancouver, Edmonton, Winnipeg, and Toronto). Mother-child pairs were then followed from birth through at least five years of age. Our analysis sample consisted of 131 mother-child pairs from an atopy-enriched subset of CHILD participants with DNAm data from cord blood and PBMCs, genotype data, prenatal and postnatal mental health measures, and important covariates. Of these, there were 119 infants with complete information for the prenatal models and 113 infants with complete information for the postnatal models. Ethical approval for human subjects research was given by the research ethics board at each study site: McMaster University, University of British Columbia, University of Manitoba, University of Alberta, and The Hospital for Sick Children. Written consent for participation was obtained from the mother at enrollment on behalf of herself and the infant.

### DNA methylation measurements

DNA methylation was measured from cord blood mononuclear cells at birth and from peripheral blood mononuclear cells at 12 months of age using the Illumina Infinium HumanMethylation450 BeadChip array. Background subtraction and color correction were performed with Illumina GenomeStudio software before data was imported to R for preprocessing. All subsequent preprocessing was performed using R version 3.5.1.

Sixty-five single nucleotide polymorphism probes were used to check concordances between paired samples and the corresponding probes were removed from the dataset. Next, probes not detected above the background or with fewer than three beads contributing to the signal in at least one sample (n = 5464), X/Y chromosome probes (n = 11,186), and poorly designed probes (n = 31,076) were removed^48^. Invariant probes as identified from a meta-analysis by Edgar et al. were also removed (n = 111,193)^49^.

Samples were determined to be outliers if detected using the *detectOutlier* function from the *lumi* package^50^. One CBMC-DNAm sample was detected as an outlier and it and its corresponding PBMC-DNAm sample were removed. The probe design bias was removed using beta-mixture quantile normalization (BMIQ)^51^ and batch effects (sentrix ID, sentrix position, and run) were removed using the *ComBat* function from the *sva* package^52^. After sampling and probe filtering, 131 samples (overlap of 113 CBMC samples and 119 PBMC samples), 307,566 CBMC probes, and 309,620 PBMC probes remained for analysis. Cell type composition estimates were calculated using the most recent reference data for cord blood^53^ and whole blood^54^. Genetic principal components were calculated to adjust for population stratification.

### Maternal stress and depression measurements

Prenatal and postnatal stress were measured using the 10-item Perceived Stress Scale (PSS)^55^. Prenatal and postnatal depression were measured using the Center for Epidemiologic Studies Depression Questionnaire (CESD)^56^. Prenatal stress and depression were measured at 18 weeks and 36 weeks of pregnancy and postnatal stress and depression were measured at six months and 12 months postpartum. For prenatal stress and depression, measurements at 18 weeks and 36 weeks were z-transformed and collapsed by addition to create composite prenatal stress and prenatal depression variables. For postnatal stress and depression, measurements at 6 months and 12 months were z-transformed and collapsed by addition to create composite postnatal stress and postnatal depression variables.

### Statistical Analysis

**Figure 1:**
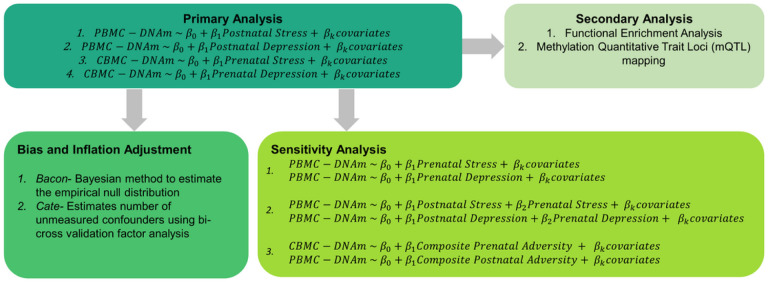
Overview of the statistical analysis pipeline. Abbreviations: PBMC-DNAm, peripheral blood mononuclear cell DNAm; CBMC-DNAm, cord bloodmononuclear cellDNAm

Our analysis pipeline consisted of a primary analysis, bias and inflation adjustment, and a series of sensitivity analyses (Fig. 1). In our primary analyses, we investigated the associations between prenatal stress (z-score) and CBMC-DNAm, prenatal depression (z-score) and CBMC-DNAm, postnatal stress and PBMC-DNAm, and postnatal depression and PBMC-DNAm. Next, we adjusted the estimates from the primary analysis for bias and inflation (e.g., due to unmeasured confounding) to assess the robustness of our primary findings. Additionally, we further explored the relationship between maternal stress and depression on infant DNA methylation at birth and the first year of life using a composite adversity score. Finally, we conducted functional enrichment analyses and methylation quantitative trait loci (mQTL) mapping as secondary analyses for all significant CpG sites to support our findings.

### Primary Analysis: Epigenome-wide Association Studies (EWAS)

We conducted EWAS for the associations between prenatal stress and CBMC-DNAm and prenatal depression and CBMC-DNAm, postnatal stress and PBMC-DNAm, and postnatal depression and PBMCDNAm. Robust linear regression was performed for each model, adjusting for the confounders described below. To correct for multiple testing, we applied the Bonferroni threshold (CBMC-DNAm models: 0.05/307566 = 1.6e-07; PBMC-DNAm models: 0.05/309620 = 1.6e-07).

### Confounding Assessment

Confounding was assessed by constructing directed acyclic graphs (DAGs). DAGs were created for each time point: prenatal stress and depression – CBMC-DNAm and postnatal stress and depression – PBMC-DNAm (Figure S1). Potential confounders were selected based on existing literature. All models were adjusted for cell-type proportions and population stratification as represented by the first five genetic principal components, which explained > 90% of the variation. Prenatal models were additionally adjusted for prenatal smoking, household income, child sex, maternal age, and study center. Postnatal models were additionally adjusted for postnatal tobacco exposure, prenatal tobacco exposure, household income, child sex, birth weight, maternal age, and study center. Prenatal tobacco exposure was ascertained at the 18th week of pregnancy and was dichotomized into a binary variable (0 = zero cigarettes smoked per day, 1 = at least 1 cigarette smoked per day). Postnatal tobacco exposure was ascertained at 3, 6, and 12 months postpartum using the average number of cigarettes smoked in the household per day (Table S1). Total postnatal tobacco exposure scores were calculated as the weighted average across the three time points.

### Bias and Inflation Adjustment

To adjust for bias due to unmeasured and residual confounding, we implemented the *bacon* and *cate* methods for bias adjustment. *Bacon* adjusts for inflation by utilizing a Bayesian method to estimate the empirical null distribution^57^. *Cate* adjusts for unmeasured confounding by first estimating the number of unmeasured confounders using bi-cross validation factor analysis and then correcting for this bias^58^.

### Sensitivity Analyses

We conducted three sensitivity analyses to assess the robustness of our primary analysis. First, to validate the robustness of our findings across exposure time points, we investigated the associations between prenatal stress and depression and infant DNA methylation (PBMC-DNAm) at 12 months of age. Second, we adjusted for prenatal stress in the postnatal stress model and for prenatal depression in the postnatal depression model to control for possible confounding by prenatal exposure. Multicollinearity between covariates was evaluated by calculating the variance inflation factors for each predictor (Table S2). Finally, to investigate the cumulative effects of stress and depression, we created a composite adversity score for each time point. For the prenatal adversity score, prenatal stress and depression were z-transformed and collapsed. Similarly, for the postnatal adversity score, postnatal stress and depression were z-transformed and collapsed.

### Functional Enrichment Analysis

To support our findings, we conducted follow-up analyses for all significant CpG sites. To identify potential biological pathways that may be altered by differential DNAm, we conducted gene ontology functional enrichment analysis using the *gometh* function of the *missMethyl* package^59^. We utilized both the Gene Ontology (GO) and Kyoto Encyclopedia of Genes and Genomes (KEGG) gene set collections available from the R package. *Gometh* identifies GO terms and KEGG pathways that are overrepresented among genes containing differentially methylated CpG sites

### Methylation Quantitative Trait Loci (mQTL) Mapping

To quantify the potential genetic influence on DNAm levels at significant CpG sites, we identified mQTLs using the GoDMC API^60^. GoDMC is a database comprising of mQTLs from over 32,000 participants from 36 cohorts using the 450K BeadChip. We filtered the results by a stringent p-value threshold of 1e-14, as the authors recommended.

## Results

### Study population characteristics

This analysis sample included 131 infants with DNAm data at birth and 12 months, genotype data, and other relevant covariates, with a subset of 119 infants and 113 infants that had complete cases for the prenatal and postnatal models, respectively (Table 1). This cohort is comprised primarily of children from higher socioeconomic backgrounds, with 45% of the population having a household income greater than $100,000 (CAD). Less than 4% of mothers smoked during pregnancy. The median maternal depression and stress scaled scores were generally low at both antenatal and postpartum time points. The median stress scaled score increased postpartum (−0.1 to 0.03), while the median depression scaled score decreased postpartum (−0.4 to −0.6).

### Prenatal stress and depression and DNAm in newborns

**Figure 2:**
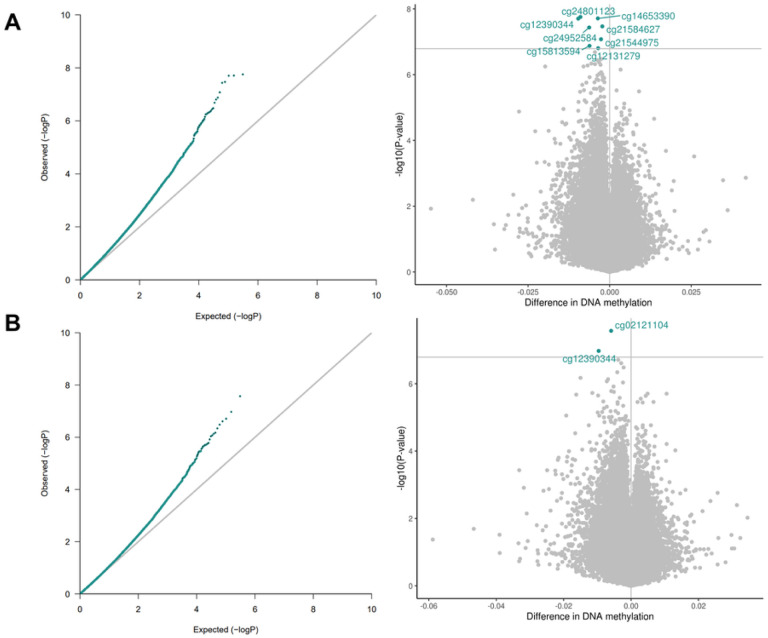
QQ and volcano plots for the associations between 2A. prenatal stress and CBMC-DNAm (lambda=1.15) and 2B. prenatal depression and CBMC-DNAm (lambda =1.01). Models were adjusted for prenatal tobacco exposure, household income, child sex, maternal age, study center, genetic principal components, and cell type proportions. Bonferroni threshold=1.6E-07.

Our analysis pipeline consisted of a primary analysis, bias and inflation adjustment, and a series of sensitivity analyses (Fig. 1). We identified a significant association between prenatal stress and differential newborn CBMC-DNAm at eight CpG sites and between prenatal depression and differential PBMC-DNAm at two CpG sites (Fig. 2; Table 2; Figures S2-S3). One CpG site (cg12390344; *LAMA3*) exhibited significantly differential DNAm for both prenatal stress (β=−9.64E-03, *P* = 1.98E-08) and prenatal depression (β=−9.61E-03, *P* = 1.06E-07). Beta estimates for prenatal stress and depression were correlated for CpG sites with p-values less than 5.0E-04 (Figure S4). After adjusting effect estimates for inflation with *bacon*¸ none of the eight CpG sites in association with prenatal stress remained significant and only one of 11 CpG sites remained significant in association with prenatal depression (cg02121104 (*EIF2B2*)). The remaining CpG sites exhibited suggestive p-values (suggestive threshold = 1E-05) (Table S3; Figure S5). Using *cate* to adjust for inflation, no CpG sites remained significant or exhibited suggestive p-values for either prenatal stress or depression (Table S3; Figure S6).

### Postnatal stress and depression and DNAm in infants

**Figure 3:**
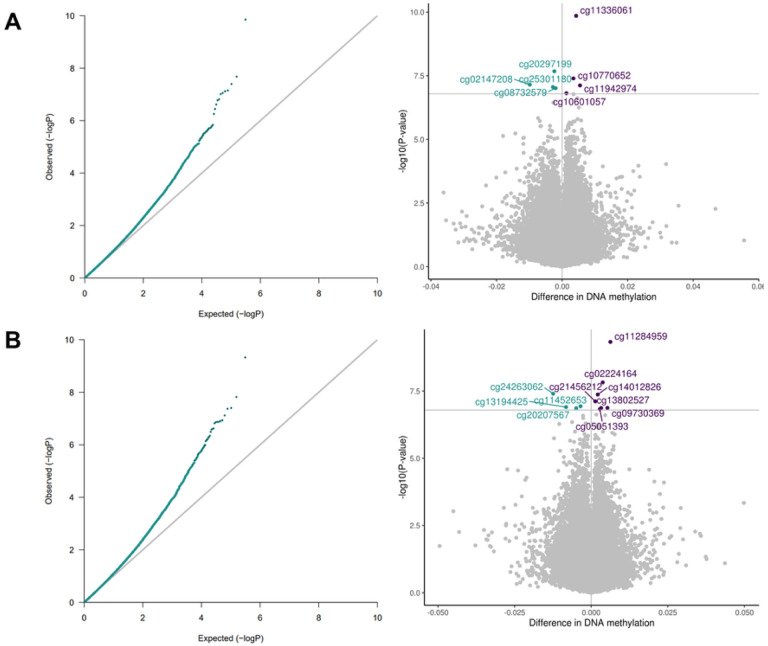
QQ and volcano plots for the associations between 3A. postnatal stress and PBMC-DNAm (lambda=1.02) and 3B. postnatal depression and PBMC-DNAm (lambda = 1.03). Models were adjusted for prenatal tobacco exposure, postnatal tobacco exposure, household income, child sex, maternal age, birth weight, study center, genetic principal components, and cell type proportions. Bonferroni threshold=1.6E-07. Blue= negative effect estimate; purple= positive effect estimate.

We identified a significant association between postnatal stress and differential child PBMC-DNAm at 12 months of age at eight CpG sites and between postnatal depression and differential PBMC-DNAm at 11 CpG sites (Fig. 3; Table 3; Figures S7-S8). There was no overlap between postnatal stress and depression CpG sites. Beta estimates for postnatal stress and depression were correlated for CpG sites with p-values less than 5.0E-04 (Figure S9). After adjusting with *bacon*, six of the eight CpG sites remained significant in association with postnatal stress and the other two CpG sites exhibited suggestive p-values. For postnatal depression, five of the 11 CpG sites remained significant in association with postnatal depression and the other 6 CpG sites exhibited suggestive p-values (Table S3; Figure S10). With *cate*, two CpG sites (cg10770652 (*BRD2*); cg25301180 (*ERC2*)) were suggestive in association with postnatal stress and one CpG site (cg05051393 (*ASF1A*)) was significant in association with postnatal depression (Table S3; Figure S11).

### Sensitivity Analyses

#### Robustness across different exposure timepoints

After investigating whether prenatal stress and depression were associated with CBMC-DNAm in newborns, we additionally assessed whether prenatal stress and depression were also associated with PBMC-DNAm at 12 months of age and whether there was overlap between both timepoints. A significant association between prenatal stress and differential PBMC-DNAm was found at four CpG sites and between prenatal depression and differential PBMC-DNAm at eight CpG sites (Table S4; Figure S12). For one PBMC-DNAm CpG site, we found robust associations across both exposure time windows (i.e., pregnancy/prenatal and 1st year of life). CpG site cg09730369 (*HYMAI; PLAGL1*) exhibited significantly differential PBMC-DNAm for both prenatal depression (β = 7.33E-03, *P* = 1.79E-12) and postnatal depression (β = 5.29E-03, *P* = 1.34E-07). Beta estimates across prenatal CBMC-DNAm- and postnatal PBMC-DNAm-associated CpG sites were also correlated (Figure S13; Figure S14). Additionally, we identified robust associations across prenatal stress and depression and PBMC-DNAm at one CpG site. This CpG site (cg03927037 (*ARGHAP20*)) exhibited significantly differential PBMC-DNAm for both prenatal stress (β=−3.61E-03, *P* = 6.52E-08) and prenatal depression (β=−3.56E-03, *P* = 4.42E-08).

#### Adjusting for prenatal stress or depression in the postnatal models

Most of the significant CpG sites from the main analysis were still either significant (cg10601057 (*CAB39*) and cg10770652 (*BRD2*) for postnatal stress; cg11284959 (intergenic) and cg11452653 (*PIP5K1C*) for postnatal depression) or at least suggestive (*P* < 0.05 for all but two CpG sites) after adjusting for prenatal stress or depression in the postnatal models (Table S5). The VIFs were not indicative of problematic multicollinearity (Table S2), but the results should still be interpreted with caution given the moderately high Pearson correlations between the two time points (prenatal and postnatal stress: 0.56; prenatal and postnatal depression: 0.65).

##### Using a combined adversity score to explore the cumulative effects of stress and depression on DNAm

The correlation between prenatal stress and depression (r = 0.82) and postnatal stress and depression (r = 0.86) was quite high. There was substantial overlap between the combined adversity score models and the separate stress and depression models from the main analysis. Two CpG sites were significantly associated with prenatal adversity and these CpG sites were also significantly associated with prenatal depression (cg12390344 (*LAMA3*)) and prenatal stress (cg12390344 (*LAMA3*) and cg21544975 (*GPR133*)) (Figure S15-S16, Table S6a). The remaining six CpG sites significantly associated with prenatal stress and one CpG site significantly associated with prenatal depression were at least suggestive in the prenatal adversity score model (all p-values < 2.85E-05). Seven CpG sites were significantly associated with postnatal adversity and six of these sites were also significantly associated with either postnatal stress (three CpG sites) or postnatal depression (three CpG sites) (Figure S17-S18, Table S6b). The CpG site that was not significantly associated with postnatal stress or depression was suggestive of an association with postnatal stress and depression (Table S7; cg23102197 (*TRIM49*); stress: p-value = 4.64E-06; depression: p-value = 1.02E-06). The remaining CpG sites were all at least suggestive of an association in the postnatal adversity score model (all p-values < 7.78E-05). Among overlapping CpG sites, the magnitude of effect was consistently stronger with the combined adversity score in comparison to the individual stress and depression models (Table S6a-S6b).

### Secondary Analyses

#### Functional Enrichment Analysis

After correction for multiple testing (FDR < 0.05), we did not identify any GO terms or KEGG pathways with an overrepresentation of genes containing significantly, differentially methylated CpGs that would indicate an enriched biological pathway. The top GO terms and KEGG pathways for each model are included in the supplement (Table S8a and Table S8b).

#### Methylation Quantitative Trait Loci (mQTL) Mapping

Of the eight CpG sites identified in association with prenatal stress and CBMC-DNAm, four (cg12390344, cg15813594, cg21584627, cg21584627) were associated with at least one mQTL (Table S9). Both CpG sites (cg02121104, cg12390344) identified in association with prenatal depression were associated with at least 1 mQTL. Of the 8 and 11 PBMC-DNAm CpG sites identified in association with postnatal stress and postnatal depression respectively, four were associated with at least one mQTL for both postnatal stress (cg12390344, cg11336061, cg20297199, cg25301180) and depression (cg11452653, cg13194425, cg13802527, cg20207567).

## Discussion

In this prospective cohort, we identified significant associations between maternal psychosocial stress and differential DNA methylation in the first year of life. Maternal stress and depression were associated with variations in the CBMC and PBMC methylome for both prenatal and postnatal exposures. Additionally, cumulative stress and depression was associated with similar variations in CBMC- and PBMC-DNAm yet demonstrated larger effect estimates. In our primary analyses we identified eight CpGs significantly associated with prenatal stress and 11 CpGs associated with prenatal depression. Additionally, eight CpGs were significantly associated with postnatal stress and 11 CpGs were associated with postnatal depression.

While maternal mental health has been extensively studied in association with childhood outcomes, investigations of the potential epigenetic mechanisms remain sparse, and findings have been inconsistent. For example, large-scale meta-analyses have not been able to identify replicable evidence of an association due to reasons such as population heterogeneity and differences in stress/depression measures that were used. One meta-analysis investigating the association between prenatal stress (composite stress score calculated from four stress domains) and cord blood DNAm (450K) did not find any evidence of an association (N = 1740)^39^. In contrast, another meta-analysis from the Pregnancy and Childhood Epigenetics (PACE) consortium investigating the association between prenatal stress (composite stress score calculated from five stress domains) and cord blood DNAm (450K and EPIC) did find evidence of an association at five CpG sites (N = 5496)^38^. However, none of the identified CpG sites were significant in our study (Table S10a). Large-scale meta-analyses have yet to be conducted for the associations between prenatal depression, postnatal stress, postnatal depression, and child DNA methylation. Several EWAS of prenatal depression and CBMC-DNAm have identified significant associations, however, none of these associations were replicated in our study^32,33,35^(Table S10b). For example, an EWAS that investigated the association between prenatal depression (Edinburgh Postnatal Depression Scale (EPDS) and Beck Depression Inventory (BDI-II)) and cord blood DNAm (N = 248) in a South African birth cohort identified one significant CpG site after bias-adjustment, however, this was not replicated in our study^33^. To the best of our knowledge, this is one of the first EWAS investigating the associations between postpartum stress and depression and DNAm and therefore we are unable to compare our results to previous findings for these analyses.

Maternal stress and depression have been hypothesized to exert their effects through multiple biological pathways such as oxidative stress, cortisol transmission through breastmilk, and the HPA axis^6,22,43^. There was substantial overlap between the combined adversity score models and the individual stress and depression models. The magnitude of effect for overlapping CpG sites was consistently larger among the combined adversity score models both prenatally and postnatally, which may indicate that cumulative stressors have a stronger effect on differential DNAm than either stress or depression alone.. Nevertheless, due to the small sample size and high correlation between the stress and depression measures in this cohort, future studies should test the replicatability of these findings and further try to disentangle the biological mechanisms between stress and depression and their cumulative effects.

While the bias-adjustment with *bacon* was generally robust with our main analysis, adjusting with the cate method yielded no significant or suggestive results. However, with our relatively small sample size and comprehensive list of included covariates *cate*, which adds additional surrogate variables as covariates, may be slightly conservative with the potential of losing true positive signals. Three CpG sites exhibited robust suggestive or significant p-values across the unadjusted and bias-adjusted estimates for the postnatal models (postnatal stress: cg10770652 (*BRD2*); cg25301180 (*ERC2*); postnatal depression: cg05051393 (*ASF1A*)). *BRD2* (bromodomain-containing protein 2) is a transcriptional regulator that plays a role in nucleosome assembly, DNA damage repair, and chromatin remodeling^61^. Mutations in *BRD2* have been implicated in juvenile myoclonic epilepsy and there is some evidence from animal models that exposure to maternal stress in combination with other teratogens may be associated with comorbid ASD and epilepsy^62,63^. Additionally, an epigenome-wide meta-analysis from the PACE consortium (N = 2190) identified differential DNAm at this gene in association with general psychopathology in school-age children^64^. *ERC2* (ELKS/RAB6-interacting/CAST family member 2) is a gene that is primarily expressed in the brain and plays a role in regulating neurotransmitter release^65^. A genome-wide association study identified *ERC2* as a locus that may induce neuronal excitability in association with febrile seizures^66^. Additionally, differential cord-blood DNAm at *ERC2* was associated with ADHD symptoms in school-age children^67^. *ASF1A* (anti-silencing function 1A histone chaperone) is a histone chaperone that is involved in chromatin assembly during DNA replication and repair. *ASF1A* is regulated by tousled-like kinases, a family of serine-threonine kinases that are involved in many regulatory functions including DNA replication and repair, chromatin structure, and genomic and epigenomic stability^68^. Dysfunction in TLK-regulated pathways has been associated with neurodevelopmental disorders such as ASD^69^. Further research is necessary to determine whether differential DNAm at *BRD2, ERC2, or ASF1A* mediate the relationship between maternal stress and neuropsychiatric outcomes in children.

A few CpG sites overlapped between time points and exposures. One CpG site exhibited differential CBMC-DNAm in association with both prenatal stress and depression (cg12390344 (*LAMA3*)). Additionally, cg03927037 (*ARHGAP20*) was differentially methylated in PBMC-DNAm in association with both prenatal stress and depression. Finally, cg09730369 (*HYMAI; PLAGL1*) was differentially methylated in PBMC-DNAm in association with both prenatal and postnatal depression. The gene *LAMA3* (laminin subunit alpha 3) is involved in cell growth, motility, and adhesion^70^. It is primarily expressed in the epidermis and pancreatic endocrine cells and has been associated with diseases such as atopic dermatitis and pancreatic cancer^71,72^. *ARHGAP20* (RHO GTPase Activating Protein 20) is involved in neurite outgrowth, differentiation, and maturation, however, not many studies have investigated this gene in association with disease^73^. Overexpression of *HYMAI* and *PLAGL1*, maternally imprinted genes, has been strongly associated with 6q24-related transient neonatal diabetes mellitus (6q24-TNDM), a rare type of diabetes that presents at birth, resolves after the first year of life and may recur in adolescence or adulthood^74,75^. Maternally imprinted genes are genes in which the maternal allele is epigenetically suppressed, and the paternal allele is epigenetically expressed through DNA and histone methylation during gametogenesis^76^. Because only one allele is expressed, imprinted genes are especially vulnerable to mutations and epigenetic changes due to environmental perturbations and can subsequently affect fetal growth and development^77–79^. One study investigated the mediation of maternal prenatal depression and birth weight by cord blood DNAm of *PLAGL1* (N = 922)^80^. While no evidence was found of mediation or an association with prenatal depression, they did identify significantly increased methylation (3.6%) at the *PLAGL1* differentially methylated region among high birth weight infants. Furthermore, another candidate gene study that investigated the mediation of maternal prenatal stress and preterm birth by cord blood DNAm of *PLAGL1* (N = 537) also did not find evidence of mediation or an association with maternal stress at this gene^81^. Larger studies and mediation analyses should be conducted to further investigate potential associations between maternal mental health and differential methylation of *HYMAI/PLAGL1* and its potential role in 6q24-TNDM.

Our study has a few limitations. First, we were unable to replicate any of our findings in previous studies due to a lack of comparability across studies. Studies that have been conducted on prenatal depression and stress use various measures of depression and stress and therefore may capture different constructs. Additionally, using cord blood and peripheral blood mononuclear cell tissue which are largely missing granulocytes, rather than unmanipulated cord or peripheral whole blood which most studies typically use may also in part explain challenges in replicating our results. Additionally, while the stress response is physiologically systematic, DNAm changes that may be exerted by maternal stress and depression may not be fully captured by blood tissue alone and could also be exerting effects in another relevant tissue such as brain^82^. Next, this study had a small sample size for both the prenatal (n = 119) and postnatal (n = 113) analyses, which likely limited our statistical power to detect associations.. Furthermore, we did not adjust for maternal intake of anti-depressant and anti-anxiety medications, a potentially important confounder. Another potential limitation is the small-magnitude effect sizes, which is common and expected in studies of early-life exposures^83^. However, even small changes in DNAm could cause differences in transcriptional regulation of gene expression^83^. Additionally, many studies of early-life exposures, such as maternal smoking during pregnancy, have found consistently small yet robust effect sizes, suggesting that small changes in DNAm may persist across populations and throughout the life-course^83^. More studies of prenatal mental health exposures need to be conducted to determine the generalizability and biological relevance of these small effect sizes. Lastly, several of our significant CpG sites were associated with at least one known mQTL, an indicator of the genetic influence on DNAm^60^. However, only a proportion of variation in DNAm is due to genetic effects. Further, the joint effect of environmental factors and single nucleotide polymorphisms (SNPs) may have a larger association with differential DNAm than SNPs alone^84,85^.

Despite these limitations, our study contributes to the sparse literature on maternal mental health and child DNAm. To our knowledge, this is one of the first EWAS to investigate the association between postpartum stress and depression and child DNAm. Additionally, we conducted a bias analysis to assess the effects of unmeasured confounding. Finally, we were able to look at multiple time points of stress, depression, and DNAm, which allowed us to investigate the possible effects of maternal mental health on DNAm across the child’s first year of life, a critical window for development.

In summary, these findings suggest that prenatal and postnatal stress and depression are associated with epigenomic changes in the fetus and the first year of life. Larger studies need to be conducted to replicate our findings and to investigate whether changes in DNAm in response to maternal mental health problems increase the risk of adverse birth and childhood outcomes.

## Figures and Tables

**Table 1:** Study population characteristics for the total population, prenatal sample, and postnatal sample.

	Overall population	Prenatal Models	Postnatal Models
**N**	131	119	113
**Child Sex (%)**			
Male	75 (57.2%)	68 (57.1%)	63 (55.8%)
Female	56 (42.7%)	51 (42.3%)	50 (44.2%)
**Maternal Age (yrs) (median [IQR])**	33 [30, 37]	33 [30, 37]	33 [30, 37]
**Prenatal Smoking (%)**			
No	126 (96.2%)	116 (97.5%)	110 (97.3%)
Yes	5 (3.8%)	3 (2.5%)	3 (2.7%)
**Postnatal Smoking (median [IQR])** ^1^	0 [0,2.5]	-	0 [0,0]
Postnatal Smoking Yes (%)			
3 months	13 (9.9%)	-	11 (9.7%)
6 months	13 (9.9%)	-	12 (10.6%)
12 months	17 (13.0%)	-	15 (13.3%)
**Household Income**			
Less than $60,000	23 (17.6%)	23 (17.6%)	22 (19.5%)
Between $60,000 and $100,000	37 (28.2%)	37 (28.2%)	34 (30.1%)
Greater than $100,000	59 (45.0%)	59 (45.0%)	57 (50.4%)
Missing	12 (9.2%)		
**Birth Weight (g) (median [IQR])**	3629 [3255,3850]	-	3620 [3260,3880]
**Study Center (%)**			
Edmonton	32 (24.4%)	30 (25.2%)	30 (26.5%)
Toronto	40 (30.5%)	36 (30.3%)	35 (31.0%)
Vancouver	21 (16.0%)	21 (17.6%)	20 (17.7%)
Winnipeg	38 (29.0%)	32 (26.9%)	28 (24.8%)
**Cell Type Proportions (mean (SD))**			
CD8-positive T Cells	-	0.08 (0.05)	0.24 (0.07)
CD4-positive T cells	-	0.31 (0.10)	0.38 (0.07)
Natural Killer Cells	-	0.08 (0.06)	0.02 (0.04)
B Cell	-	0.11 (0.05)	0.25 (0.07)
Monocytic Cell	-	0.10 (0.07)	0.11 (0.07)
Granulocytes^2^	-	0.24 (0.13)	0.03 (0.03)
Nucleated Red Blood Cells	-	0.09 (0.13)	-
**Prenatal Stress Scaled Score (median [IQR])**	−0.1 [−1.3,1.3]	−0.1 [−1.3,1.0]	-
**Prenatal PSS Raw Score (median [IQR])**			
18 weeks	11.0 [6.0,17.0]	12.0 [6.0, 16.5]	-
36 weeks	11.0 [7.0,17.0]	11.0 [7.0, 17.0]	-
**Prenatal Depression Scaled Score (median [IQR])**	−0.4 [−1.3,0.9]	−0.4 [−1.3,0.9]	-
**Prenatal CES-D Raw Score (median [IQR])**			
18 weeks	6.0 [3.0,11.0]	6.0 [3.0, 11.0]	-
36 weeks	8.0 [3.5,12.0]	7.0 [3.5, 12.0]	-
**Postnatal Stress Scaled Score (median [IQR])**	0.03 [−1.1,1.1]	-	−0.10 [−3.0,1.2]
**Postnatal PSS Raw Score (median [IQR])**			
6 months	11.0 [5.0,16.0]	-	10.0 [5.0, 16.0]
12 months	12.0 [7.0,17.0]	-	12.0 [7.0, 17.0]
**Postnatal Depression Scaled Score (median [IQR])**	−0.6 [−1.2,0.7]	-	−0.6 [−1.3,0.7]
**Postnatal CES-D Raw Score (median [IQR])**			
6 months	4.0 [1.0,11.5]	-	4.0 [1.0, 11.0]
12 months	6.0 [2.0,13.0]	-	5.0 [2.0, 12.0]
**Prenatal Adversity Composite Score (median [IQR])**	−0.1 [0.8,0.6]	−0.09 [−0.8,0.6]	-
**Postnatal Adversity Composite Score (median [IQR])**	−0.2 [−0.7,0.5]	-	−0.3 [−0.7,0.6]

1 Weighted average of postnatal smoking across all three timepoints. Categorical data of postnatal smoke exposure across the three timepoints can be found in Table S1.

2 For the whole blood reference, only neutrophil cell type estimates are provided.

**Table 2:** Effect sizes and p-values of significant CpG sites for prenatal stress and depression and CBMC-DNAm in newborns.

Exposure	CpG (Gene)	Mean effect size^1^ (SE)	P value	Position	Chr
	cg12131279 *(COL20A1)*	−3.53E-03 (6.73E-04)	1.57E-07	61951439	20
Prenatal Stress	cg12390344 *(LAMA3)*	−9.64E-03 (1.72E-03)	1.98E-08	21270348	18
	cg14653390	−3.63E-03 (6.46E-04)	1.95E-08	2930315	1
	cg15813594 *(EGFLAM)*	−6.17E-03 (1.17E-03)	1.33E-07	38445563	5
	cg21544975 *(GPR133)*	−2.64E-03 (4.93E-04)	8.37E-08	131526516	12
	cg21584627	−2.25E-03 (4.07E-04)	3.40E-08	92649211	11
	cg24801123 *(ADCY1)*	−8.98E-03 (1.59E-03)	1.76E-08	45615503	7
	cg24952584 *(LRRC15)*	−6.28E-03 (1.14E-03)	3.69E-08	194080771	3
Prenatal Depression	cg02121104 *(EIF2B2)*	−5.92E-03 (1.06E-03)	2.69E-08	75470314	14
	cg12390344 *(LAMA3)*	−9.61E-03 (1.81E-03)	1.06E-07	21270348	18

1 The mean effect size represents the difference in DNA methylation according to the range of the distribution of β values which are on a 0 to 1 scale.

**Table 3:** Effect sizes and p-values of significant CpG sites for postnatal stress and depression and PBMC-DNAm at 12 months of age.

Exposure	CpG (Gene)	Mean effect size^1^ (SE)	P value	Position	Chr
Postnatal Stress	cg02147208	−9.83E-03 (1.82E-03)	7.06E-08	21662648	6
	cg08732579 *(RILPL1)*	−1.98E-03 (3.71 E-04)	9.71E-08	123957442	12
	cg10601057 *(CAB39)*	1.34E-03 (2.55E-04)	1.54E-07	231639268	2
	cg10770652 *(BRD2)*	3.47E-03 (6.33E-04)	4.01E-08	32945243	6
	cg11336061 *(RUNX3)*	4.30E-03 (6.70E-04)	1.40E-10	25254149	1
	cg11942974	5.48E-03 (1.02E-03)	7.60E-08	145092423	2
	cg20297199 *(BMP4)*	−2.35E-03 (4.20E-04)	2.10E-08	54422775	14
	cg25301180 *(ERC2)*	−2.78E-03 (5.19E-04)	8.79E-08	56502091	3
Postnatal Depression	cg02224164 *(MARS)*	3.77E-03 (6.67E-04)	1.51E-08	57883315	12
	cg05051393 *(ASF1A)*	2.81E-03 (5.35E-04)	1.51E-07	119228846	6
	cg09730369 *(HYMAI;PLAGL1)*	5.29E-03 (1.00E-03)	1.34E-07	144328421	6
	cg11284959	6.26E-03 (1.01E-03)	4.72E-10	86205515	5
	cg11452653 *(PIP5K1C)*	−3.49E-03 (6.58E-04)	1.16E-07	3659919	19
	cg13194425 *(FGF11)*	−8.20E-03 (1.55E-03)	1.25E-07	7341936	17
	cg13802527 *(C9orf80)*	3.23E-03 (6.13E-04)	1.35E-07	115481039	9
	cg14012826 (ZNF416)	2.13E-03 (3.88E-04)	4.27E-08	58083387	19
	cg20207567 (NEIL2)	−4.88E-03 (9.26E-04)	1.38E-07	11626510	8
	cg21456212 (ATP11A)	1.34E-03 (2.49E-04)	7.69E-08	113359836	13
	cg24263062 (EBF4)	−0.01 (2.26E-03)	3.97E-08	2730191	20

1 The mean effect size represents the difference in DNA methylation according to the range of the distribution of β values which are on a 0 to 1 scale.

Abbreviations: PBMC-DNAm, peripheral blood mononuclear cell DNAm; CBMC-DNAm, cord blood mononuclear cell DNAm
